# Effects of CpG Methylation on Recognition of DNA by the Tumour Suppressor p53

**DOI:** 10.1016/j.jmb.2008.11.054

**Published:** 2009-02-13

**Authors:** Miriana Petrovich, Dmitry B. Veprintsev

**Affiliations:** 1 MRC Centre for Protein Engineering, Cambridge CB2 0QH, UK; 2 MRC Laboratory of Molecular Biology, Cambridge CB2 0QH, UK

**Keywords:** p53, DNA methylation, DNA binding, fluorescence anisotropy, CpG

## Abstract

Methylation of DNA is one of the mechanisms controlling the expression landscape of the genome. Its pattern is altered in cancer and often results in the hypermethylation of the promoter regions and abnormal expression of tumour suppressor genes. Methylation of CpG dinucleotides located in the binding sites of transcription factors may contribute to the development of cancers by preventing their binding or altering their specificity. We studied the effects of CpG methylation on DNA recognition by the tumour suppressor p53, a transcription factor involved in the response to carcinogenic stress. p53 recognises a large number of DNA sequences, many of which contain CpG dinucleotides. We systematically substituted a CpG dinucleotide at each position in the consensus p53 DNA binding sequence and identified substitutions tolerated by p53. We compared the binding affinities of methylated *versus* non-methylated sequences by fluorescence anisotropy titration. We found that binding of p53 was not affected by cytosine methylation in a majority of cases. However, for a few sequences containing multiple CpG dinucleotides, such as sites in the *RB* and *Met* genes, methylation resulted in a four- to sixfold increase in binding of p53. This approach can be used to quantify the effects of CpG methylation on the DNA recognition by other DNA-binding proteins.

## Introduction

Methylation of DNA is a powerful epigenetic mechanism used by eukaryotes to control the expression landscape of the genome. It is involved in cell differentiation, X chromosome inactivation, and regulation of transcription. Methylation occurs on the pyrimidine ring of cytosine, resulting in 5-methylcytosine. In mammals, it is almost exclusively the CpG dinucleotide that is methylated. Aberrant DNA methylation is associated with a large number of human diseases including cancer.^1–4^ Methylation of DNA is associated with repression of transcription. Repression can be achieved by preventing sequence-specific transcription factors binding their specific binding sites^5^ or, alternatively, by proteins that recognise methylated DNA inducing a change in the state of the chromatin and preventing transcriptional machinery from accessing the promoter region.^6^ Reported examples of DNA-binding proteins affected by the methylation state of DNA include MLTF^5^ and CTCF,^7^ which do not bind methylated DNA. Other documented examples include methyl-CpG binding proteins, which specifically recognise methylated DNA (reviewed by Klose and Bird).^8^ Yet other transcription factors, such as Sp1, are insensitive to methylation in their binding sites.^9^ There are an increasing number of examples of methylation of DNA not completely silencing transcription, but rather attenuating the rate of transcription or playing a role in the normal regulation of transcription.^10^ As a more detailed picture of the DNA methylation pattern of the genome is emerging, it is important to be able to characterise the effects of CpG methylation on protein–DNA interactions.

The tumour suppressor protein p53 is a transcription factor that plays a key role in response to carcinogenic stress and prevention of tumour development.^11,12^ Mutations in p53 are associated with 50% of all human cancers.^13,14^ p53 recognises a 20-bp DNA sequence consisting of two repeats of 5′-RRRCWWGYYY-3′ (where R = A or G; Y = C or T; W = A or T) separated by 0–13 bp.^15^ p53 recognises a large number of sequences that deviate from this consensus site definition,^16^ many of which contain CpG dinucleotides. The promoter regions of transcriptional targets of p53, such as *gadd45*, *maspin* or *bax*, tumour suppressor proteins themselves, are often hypermethylated in cancers.^17–19^ We estimated that about 20% of the hundreds of thousands of putative p53 binding sites contain one or more CpG dinucleotides.^20^ The methylation of DNA may, therefore, significantly affect the tumour suppressor activity of p53.

Using fluorescence anisotropy titrations, we studied the effects of CpG methylation on the recognition of DNA by the tumour suppressor p53. In order to address the whole range of DNA sequences recognised by p53, we systematically substituted a CpG dinucleotide at each position in the consensus p53 DNA binding sequence. Using an automation platform to multiplex titration experiments,^20^ we found that a CpG dinucleotide can be tolerated at some but not all positions of the p53 binding sequence. We compared the binding affinities of methylated *versus* non-methylated sequences containing a CpG dinucleotide at every position in the p53 binding site. We also studied the effects of methylation of only one DNA strand and the effects of having multiple methylated CpG dinucleotides in the binding site. This method is sensitive to changes in binding affinity caused by methylation of CpG and quantifies the difference. While the affinity of p53 depended on the exact sequence used, the changes attributed specifically to methylation were modest. However, the binding of p53 was markedly stronger to methylated DNA if multiple CpG dinucleotides were present in the binding site. Our results indicate that p53 recognises its binding sites containing methylated CpG dinucleotides with affinity and specificity comparable to that of non-methylated sites, but that it can be sensitive to methylation for a small fraction of binding sites.

## Results

### Nucleotide design and binding experiments

**Fig. 1:**
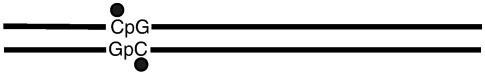
To access the whole variety of DNA sequences recognised by p53, we systematically introduced CpG or 5-methyl-CpG at every possible position in the sequence.

p53 recognises a large number of 20-bp DNA sequences consisting of two repeats of RRRCWWGYYY separated by 0–13 bp.^15^ Recent data suggest that this definition is too strict, and that p53 recognises a set of sequences that deviate from such a definition.^16,20^ A CpG dinucleotide may occupy any position within the p53 binding site. To address the potential impact of CpG methylation on the whole range of DNA sequences p53 recognises, we systematically substituted a CpG dinucleotide and a methylated CpG dinucleotide at each position in the consensus p53 DNA binding sequence. We chose a 26-bp sequence, CGCGGACATGTCCGGACATGTCCCGC, as a reference sequence. It consists of two identical copies of the GGACATGTCC half-site that is representative of a consensus sequence, RRRCWWGYYY, flanked by CGC triplets to improve the annealing properties of the oligonucleotide while minimising self-annealing. To assess the effect of methylation on both strands of DNA, we inserted methylated CpG on both strands (Fig. 1). Since the variation of the sequence affects the affinity, we used the identical unmethylated sequence as a control for each methylated sequence (Table 1).

**Fig. 2:**
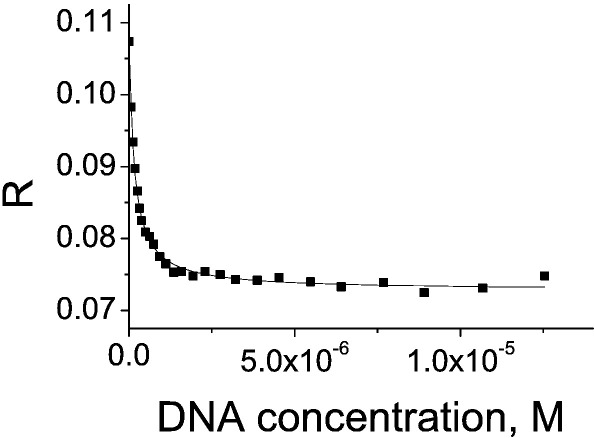
Fluorescence anisotropy *R* reflects the tumbling rate of molecules in solution. It is ideal for studying strong protein–DNA interactions, as the complex formed is larger and tumbles more slowly than does the unbound oligonucleotide. A displacement of the Alexa488-labelled reporter oligonucleotide from the complex by the unlabelled competitor oligonucleotide allows accurate measurement of the difference in the *K*_d_ between two sequences. For clarity, the data shown represent a single titration using reference sequence number 300 from Table 1. Reporter DNA sequence (20 nM) was mixed with 125 nM full-length p53 protein. Competitor DNA was added to the sample in 25 steps. Such a titration was done for every DNA sequence studied. We multiplexed our measurements to perform 96 such titrations in parallel, allowing simultaneous analysis of a number of different sequences (see Materials and Methods for details).

We measured the binding affinity for the fluorophore-labelled reporter sequence using direct fluorescence anisotropy titrations as previously described.^21^ Fluorescence anisotropy is the property of fluorescent molecules to retain the polarisation of the excitation light and reflects the tumbling rate of molecules in solution. It is ideal for studying protein–DNA interactions, as the complex formed is larger and tumbles more slowly than the unbound oligonucleotide. In subsequent experiments, we used a competition assay^20^ to improve the accuracy of determination of the difference in the affinity of the binding protein for the reporter and competitor DNA (Fig. 2). The 22-bp palindromic reporter sequence CGGACATGTCCGGACATGTCCG, consisting of a representative p53 consensus sequence flanked by a single C or G nucleotide, was labelled at the 5′ end with Alexa488 fluorophore. A shorter flanking region (1 bp compared with 3 bp for unlabelled competitor sequences) was used to increase the amplitude of the anisotropy changes in the experiment. For competition experiments, the reporter sequence was mixed with p53 protein to form a complex, and unlabelled oligonucleotide was added to the cuvette in small aliquots to compete off the reporter oligonucleotide. Analysis of the competition curve allowed accurate determination of the difference in the affinities of the two oligonucleotides. The reporter sequence is only used to monitor the binding reaction, and the comparison of affinities is between unlabelled sequences of identical length.

**Fig. 3:**
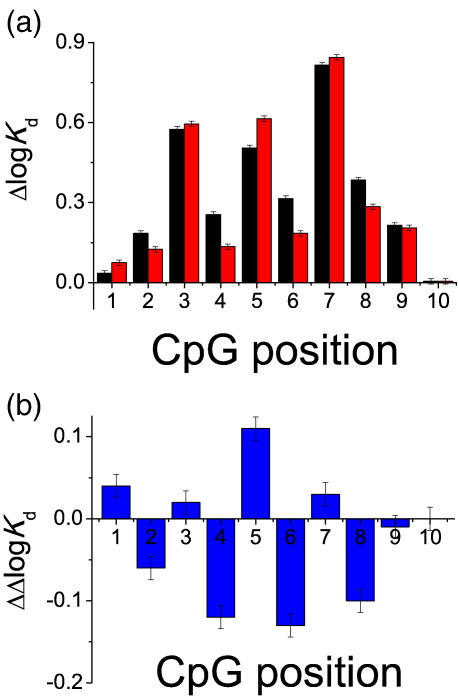
(a) Effects of CpG methylation on affinity of p53 for DNA. CpG (black) or 5-methyl-CpG (red) dinucleotide was systematically substituted into the reference sequence at positions 1–10 of the first half-site. The CpG position refers to the position of the C in the first half of the p53 binding sequence GGACATGTCC. The difference in affinity relative to the unmethylated reference sequence is shown (Δlog *K*_d_ = log *K*_d(i)_ − log *K*_d(ref)_). The log *K*_d_ of the reference sequence was − 7.55. (b) The difference in the affinity that can be attributed specifically to methylation is the difference in height of the bars at the same position, ΔΔlog *K*_d_. = Δlog *K*_d(met)_ − Δlog *K*_d(non-met)_. Introduction of CpG dinucleotide at positions 4 and 6 has small overall impact on the affinity of p53 and results in the biggest methylation-specific response. Error bars represent one standard deviation based on at least three individual titrations.

**Fig. 4:**
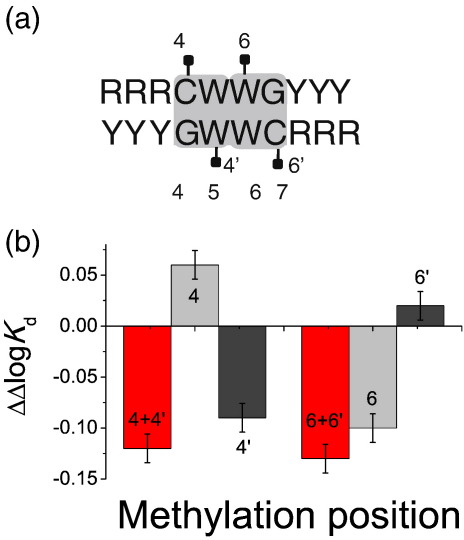
Effects of single-strand methylation on binding of p53 to DNA. (a) Schematic diagram representing a consensus sequence of a p53 half-site. (b) A change in the affinity caused by a specific methylated cytosine or a combination of cytosines in a CpG dinucleotide. Methylation of CpG dinucleotides at positions 4 and 6 has the largest effect on binding of p53. These modifications affect invariant nucleotides at positions 4 and 6′, respectively. When methylated cytosine is present on only one strand, the effects of methylation at positions 5 and 6 had a similar effect as double methylation. 4, methylation on the top strand; 4′, methylation on the bottom strand; 4 + 4′, methylation on both strands. Likewise for position 6. The log *K*_d_ of the reference sequence was − 7.55.

We compared the affinity of oligonucleotides containing single methylated and unmethylated CpG dinucleotide to the affinity of unlabelled reference sequence. The changes in the affinity caused by all possible substitutions of CpG dinucleotide within the reference oligonucleotide (Table 1) are presented in Fig. 3. Because of the symmetry of the sequence used (it is the same sequence on the complementary strand in the 5′ to 3′ direction) only the first half-site was scanned, the sequence of the second half-site being unaltered. First, we investigated if p53 binds to sequences containing CpG dinucleotide by comparing the affinity of the CpG-containing sequence to the reference sequence (Fig. 3a, black series). The substitution of the CpG dinucleotide in the reference sequence makes it suboptimal in many cases. Substitutions at positions 3, 5 and 7 resulted in a particularly large reduction of the affinity (increase in the log *K*_d_ value). These substitutions replace the invariant C and G with G and C at positions 4 and 6, respectively (Fig. 4). These results are consistent with our previous data on the sequence dependence of the affinity using single-nucleotide substitutions, as well as the consensus sequence definition.^15,16,20^ The CpG at position 5 replaces the WW (W = A or T) motif at positions 5/6. This motif is important for recognition of DNA, with the TA dinucleotide being the best both for binding and for transactivation activity.^15,16,20^

To investigate the effects of methylation, we compared the affinity of sequences containing CpG dinucleotides with identical sequences containing CpG dinucleotides methylated on both strands (Fig. 3a and b). The effects that we were able to attribute specifically to methylation were modest (Fig. 3b). The largest differences caused by methylation were measured at positions 4, 5, 6 and 8 (Fig. 3). Since substitution of the CpG dinucleotide at positions 4 or 6 preserved the invariant C and G at these positions, the reduction in the affinity caused by the sequence change was not very large. Consequently, it is more likely that methylation at these positions are physiologically relevant.

In our experiments, each CpG substitution was methylated on both strands of DNA. Methylation of the C at position 4 of the p53 half-site was accompanied by methylated C at position 5 on the complementary strand. To investigate if the effects of methylation are strand-specific, we compared the affinity of sequences containing CpG dinucleotide at positions 4 and 6 with methylated cytosine present on only one of the DNA strands with unmethylated sequence or sequence containing methylated cytosine on both strands (Fig. 4). We prepared the competitor DNA by annealing a strand containing 5-methylcytosine with a complementary non-methylated strand (see Materials and Methods). Single methylated cytosine at positions 5 (4′ in Fig. 4) and 6 (6 in Fig. 4) gave effects similar to those observed when methylated cytosine was present on both strands. The presence of methylated cytosine at positions 4 and 7 alone caused smaller changes in the affinity in the opposite direction.

**Fig. 5:**
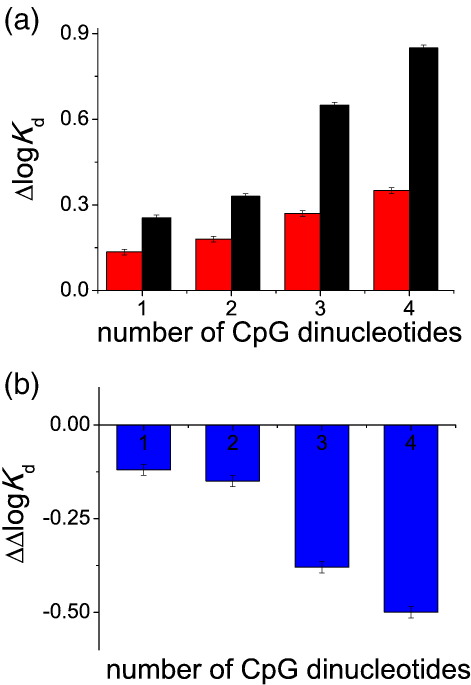
Effects of multiple methylated CpG on the affinity of p53 for DNA. (a) Multiple CpG substitution results in progressively weaker binding because of the increasingly suboptimal DNA sequence. However, if the CpG dinucleotides are methylated, the overall increase in the log *K*_d_ is smaller. (b) The difference between the affinity of the methylated and non-methylated sequence increased proportionally to the number of methylated CpG dinucleotides. The log *K*_d_ of the reference sequence was − 7.55.

Although the effects of a single methylated CpG on the affinity of p53 are relatively small, the presence of multiple CpG dinucleotides in the binding site may result in significant changes in affinity. We tested that p53 can bind to sites containing 1, 2, 3 or 4 CpG dinucleotides at positions corresponding to positions 4 or 6 of the half-site (Fig. 5a). The log *K*_d_ progressively increased with the number of CpG dinucleotide substitutions (black series). However, if the CpG dinucleotides were methylated, p53 bound DNA more strongly, and the changes caused by methylation progressively increased (Fig. 5b). Depending on the exact sequence of the whole site, sites containing multiple CpG dinucleotides can be high-affinity sites, comparable to the strong p53 binding site in the promoter regions of p21, and these bind p53 significantly more strongly in the methylated state.

## Discussion

Methylation of CpG dinucleotides is generally associated with silencing of transcription, which may be achieved by preventing binding of specific transcription factors or by inducing a global change in the structure of DNA. We set out to develop a method for quantifying the effects of CpG methylation on the binding of transcription factors and applied this method to p53. We used fluorescence anisotropy titration of a fluorophore-labelled oligonucleotide as an accurate method of measuring protein–DNA interactions in solution. We have successfully applied it before to study the mechanism of the DNA binding of p53 and to characterise its sequence specificity.^20–22^ Many transcription factors are not “strict” in their DNA-binding preferences and can bind a large number of variants of the consensus sequence. Such sequences may contain a CpG dinucleotide at various positions. First, we established how likely it is to find a CpG dinucleotide in the binding site of p53.

We compared the affinity of binding of a series of DNA sequences representing a consensus sequence with CpG dinucleotide systematically substituted at every position in the sequence. We found that substitution of CpG at some (3, 5 and 7) positions resulted in a large decrease in the affinity (Δlog *K*_d_ of 0.5 to 0.8) of such sequence to p53. Consequently, such sequences are unlikely to be active binding sites. However, substitution of CpG at other positions in the site resulted in only a small decrease of affinity (Δlog *K*_d_ of 0.05 to 0.3–0.4) and could be tolerated.

To measure the methylation-specific effects, we compared the affinity of binding of identical sequences containing CpG dinucleotides, one of which contained methylated CpG dinucleotide. In our experiments, we used synthetic 5-methylcytosine-containing oligonucleotides. This allowed us to observe effects entirely attributable to methylation, with no corrections for efficiency of modification. The effects caused by incorporation of a methylated dinucleotide were relatively modest. Taking into account the cooperativity of binding of p53 to DNA, they may result in up to a 70% increase in binding. If we combine the scale of effects caused by methylation (Fig. 3b) at specific positions with relatively small changes in affinity caused by the incorporation of the CpG in the consensus sequence (Fig. 3a), positions 4 and 6 (Δlog *K*_d_ of 0.12), followed by 8 (0.1) would emerge as the more important methylation sites.

Since each methylated CpG included 5-methylcytosine on both strands of DNA, we investigated if methylation of only one of the strands can account for the observed changes. The effects of single-methylated cytosine on one strand only in the middle of a half-site (Fig. 4) caused effects similar to those in the presence of methylation on both strands. Interestingly, methylation of the invariant cytosine at position 4 and complementary to invariant guanidine at position 7 resulted in a small decrease in the affinity rather than an increase. This indicated that the effects of individual methylation in the CpG dinucleotide are not independent at these positions and are dominated by methylation in the middle of the half-site.

The effect of a single methylated CpG is small and, taking cooperativity of binding into account, results in up to a 1.7-fold increase in the occupancy of a site under optimal conditions (for details of calculations, see Ref. 20). Multiple CpG dinucleotides in a p53 site, at these positions, can result in a greater sensitivity of p53 binding to methylation. Our measurement indicate that the effects of multiple CpG dinucleotides are additive. The more CpG dinucleotides are present in the binding sequence, the weaker p53 binds. However, if CpG dinucleotides are methylated, the affinity is partially restored. The more CpG dinucleotides there are, the more sensitive such sites become to methylation (Fig. 5). The proportion of sites containing CpG for a given transcription factor can vary from 0% to 100%. By searching a data set of all putative p53 binding sites in the genome, we estimated that about 20% of the putative p53 binding sites contained at least one CpG dinucleotide.^20^ There were almost no sites with CpG at positions 3, 5 and 7, consistent with the observation that such substitutions dramatically reduce affinity towards p53. Approximately 7% of putative p53 binding sites contain a CpG dinucleotide in position 4 or 6. Approximately 0.2% of all putative binding sites contained two CpG dinucleotides at these positions, and only 0.0016% contained three CpG dinucleotides. Such a decrease in a number of putative binding sites reflects the reduction of the affinity caused by inclusion of CpG dinucleotides in the binding site. It is not clear if any of the putative binding sites containing multiple CpGs are actually active in the cell. Unfortunately, we are far from having a complete list of all p53 binding sites, even current estimates of the number of sites varying by an order of magnitude.^23–25^ The scale of the effects caused by methylation (0.12 for one to 0.5 for four methylated CpGs; Fig. 5) is comparable to the effect of a single nucleotide polymorphism in the promoter of the *flt-1* gene (corresponding to a Δlog *K*_d_ of 0.3), which activates a non-canonical p53 binding site.^26^ While the DNA sequence rarely changes in a given cell, methylation may provide an alternative mechanism affecting the affinity of a binding site in these special but rare cases. It is not clear how many or which p53 binding sites are active in the cell and what proportion of them contains CpG dinucleotides. The whole-genome chromatin immunoprecipitation (ChIP) assay data have identified 542 and 1546 high-confidence sites,^23,27^ but extrapolations from the detailed analysis of smaller fractions of the genome suggested a larger number of 1500–3700.^24,25^ Analysis of the data set of the 95 experimentally verified p53 binding sites,^28–30^ summarised in Ref. 31, suggests that CpG dinucleotides are surprisingly common: 38 out of 95 contained at least one CpG (Table 2). In five cases (5/95), the sites contained more than one CpG dinucleotide located in positions where methylation can result in a significant change in affinity. The most dramatic example is the p53 binding site in the promoter region of the *RB* gene that contained five CpGs, of which three were in positions corresponding to positions 4 or 6 of the half-site. The product of the *RB* gene (pRb) is an inhibitor of cell cycle progression and an important tumour suppressor itself (Ref. 32 and references within). Mutations in this protein were originally identified as a cause of retinoblastoma cancer, and its inactivation is associated with a wide spectrum of human cancers. Other examples involved the p53 binding site in the promoter region of the *Met* proto-oncogene that encodes the receptor of the hepatocyte growth factor/scatter factor and implicated in the tumour invasion;^33^ *cFOS*, encoding a tumour-associated transcription factor involved in formation of activator complex 1 (AP-1); a gene for ornithine decarboxylase, *ODC1*; and a gene, *HSPA8*, encoding a constitutively expressed member of heat shock 70 protein family. There were also six more sites (*BAX*, *mdm2*, *S100A2*, *EEf1A1*, second site in *ODC1*, and *sls38*) that contained a single CpG dinucleotide in position 4 or 6. Binding of p53 to its target sites may, of course, be altered by other factors present in the cell. What can be said is that in the specific cases cited above, the methylation of CpG dinucleotides should have biological consequences.

Evidently, such sites have to be methylated in order for methylation to have functional consequences in the cell. The advent of high-throughput sequencing,^34^ combined with methylated DNA immunoprecipitation methods (MeDIP),^35,36^ holds great promise for generating genome-wide methylation maps for a variety of cell lines and tumour cells and of telling us the methylation state of a particular p53 binding site (or other transcription factors in question). The functional role of methylation could then be studied by following changes in the transcription of target genes in response to inhibition of DNA-methylation enzymes.

The effects of DNA methylation on binding are specific to every transcription factor studied so far. While binding of Sp1 and YY1 to DNA is not affected by methylation,^9,37^ other transcription factors are sensitive to methylation. The binding site for MLTF(USF) contains two CpG dinucleotides, but the binding is significantly reduced by methylation of only one of them.^5^ Binding of ETS transcription factors is also sensitive to CpG methylation, and methylation at one particular position of all the available CpGs is sufficient to reduce binding. More specifically, it is sensitive to methylation on one strand of the DNA and not the other.^37^ p53 is capable of binding to the methylated promoter region of the *DSC3* gene,^38^ which contains methylated CpG dinucleotide in the p53 binding site. Our data, collected *in vitro*, are consistent with this observation. We demonstrated that p53 binds directly to DNA sites containing a methylated CpG dinucleotide, with comparable affinity and specificity as non-methylated sites. For a small fraction of all p53 putative binding sites and a few experimentally confirmed binding sites, which contain multiple CpGs, the methylated state may bind p53 more strongly. The comparison of affinity of a consensus sequence containing methylated or non-methylated CpG dinucleotide, systematically substituted at each position, provides a convenient method for quantifying the effects of DNA methylation. Given that 70–80% of all CpG dinucleotides in the human genome are methylated,^39^ studying the effects of DNA methylation on the binding of transcription factors may contribute to our understanding of the effects caused by changes in the methylation pattern of the genome.

## Materials and Methods

We used the superstable mutant of full-length p53 containing the following mutation in the core domain: M133L/V203A/N239Y/N268D.^40,41^ These mutations increased sample stability and protein expression levels. DNA sequence encoding the full-length p53 containing stabilizing mutations was cloned into a pET24a-HLTV plasmid.^42^ It was expressed overnight in *Escherichia coli* cell strain BL21(DE3)pLysS following induction with 1 mM IPTG in 2TY medium (16 g Tryptone, 10 g yeast extract, 5 g NaCl, water to 1L) at 20 °C. The protein was purified by using standard His-tag purification protocols, followed by tobacco etch virus protease digestion. The final purification step was heparin-affinity chromatography.^42^ Purified protein was concentrated to 20–100 μM, flash-frozen in liquid nitrogen in 0.5-ml aliquots and stored at − 80 °C.

All other reagents were of the highest grade available. Synthetic DNA oligonucleotides, including nucleotides containing 5-methylcytosine (competitor DNA), and Alexa488-labelled consensus oligonucleotide (reporter DNA) were ordered from Eurogentec, Belgium. Oligonucleotide concentration was quantified by absorbance and normalised to 1 mM using an epMotion 5070 pipetting robot (Eppendorf AG, Germany) prior to annealing. Oligonucleotides were annealed by heating to 95 °C for 5 min and cooling at 1 °C/min to room temperature in a PCR block (PTC-100, MJ Research, Inc) and diluted to a final concentration of 50 μM.

DNA-binding experiments were done by fluorescence anisotropy at 25 °C in a Varian Eclipse fluorimeter.^42^ The buffer conditions for the binding experiments were 25 mM NaPi, 225 mM NaCl, 10% (v/v) glycerol, and 5 mM DTT. Total ionic strength was 286 mM. Bovine serum albumin (0.2 mg/ml) was added to buffers to minimise non-specific binding of proteins, particularly at low protein concentrations. The concentration of reporter DNA was 20 nM. For competition experiments, 20 nM reporter DNA was mixed with full-length p53 to a final concentration of 125 nM. A 25, 50 or 100 μM stock of competitor DNA was added in small aliquots to compete the reporter DNA off the complex. DNA-binding experiments were multiplexed by performing titrations on microtiter plates (Corning 3650) with a Bravo 96-channel pipetting robot (Velocity11, USA) interfaced with a Pherastar plate reader (BMG LABTECH GmbH, Germany) using a 480/520-nm fluorescence polarisation module. The instruments were controlled by manufacturer-provided software. Titrations were done at 25 °C. The sample volume during the titration was kept constant (200 μl): prior to addition of an aliquot of the competitor DNA, an equal volume of the sample was removed by aspiration. To keep the concentration of reporter DNA and protein constant, the competitor DNA stock also contained labelled reporter DNA and p53. Thus, only the concentration of the competitor DNA changes during the titration. To minimise the errors associated with handling small volumes (< 1 μL), a 2.5 μM stock of competitor DNA was used for the first part of the titration, switching to 25 μM for the second part. The samples were mixed after each addition by repeated (20 cycles) pipetting. Overall, the whole titration consisting of 26 points took 2.5 h (6 min per step, of which approximately 5 min was sample mixing and incubation prior to the measurements). Source microtiter plates were prepared using an epMotion 5070 pipetting robot (Eppendorf AG, Germany). In each experiment, 96 individual titrations were performed in parallel. Each competitor DNA sequence was represented at least three times per plate. Each plate contained the reference sequence as a control. Data were analysed using laboratory software according to a competition model.^20^

## Figures and Tables

**Table 1:** Sequences of DNA used in the study

300	CGCGGACATGTCCGGACATGTCCCGC
301	CGCCGACATGTCCGGACATGTCCCGC
m301	CGCC⁎GACATGTCCGGACATGTCCCGC
302	CGCGCGCATGTCCGGACATGTCCCGC
m302	CGCGC⁎GCATGTCCGGACATGTCCCGC
303	CGCGGCGATGTCCGGACATGTCCCGC
m303	CGCGGC⁎GATGTCCGGACATGTCCCGC
304	CGCGGACGTGTCCGGACATGTCCCGC
m304	CGCGGAC⁎GTGTCCGGACATGTCCCGC
305	CGCGGACCGGTCCGGACATGTCCCGC
m305	CGCGGACC⁎GGTCCGGACATGTCCCGC
306	CGCGGACACGTCCGGACATGTCCCGC
m306	CGCGGACAC⁎GTCCGGACATGTCCCGC
307	CGCGGACATCGCCGGACATGTCCCGC
m307	CGCGGACATC⁎GCCGGACATGTCCCGC
308	CGCGGACATGCGCGGACATGTCCCGC
m308	CGCGGACATGC⁎GCGGACATGTCCCGC
309	CGCGGACATGTCGGGACATGTCCCGC
m309	CGCGGACATGTC⁎GGGACATGTCCCGC
310	CGCGGACATGTCCGGACATGTCCCGC
m310	CGCGGACATGTCC⁎GGACATGTCCCGC
322	CGCGGACGCGTCCGGACATGTCCCGC
m322	CGCGGAC⁎GC⁎G TCCGGACATGTCCCGC
323	CGCGGACGCGTCCGGACGTGTCCCGC
m323	CGCGGAC⁎GC⁎GTCCGGAC⁎GTGTCCCGC
324	CGCGGACGCGTCCGGACGCGTCCCGC
m324	CGCGGAC⁎GC⁎GTCCGGAC⁎GC⁎GTCCCGC

C⁎ represents 5-methylcytosine. CpG dinucleotides are underlined. The sequences were generated by systematically substituting the specific dinucleotides from positions 1 to 10 in a half-site (i.e., 4–13 including 3-bp flanking region) with CpG or 5-methyl-CpG. Only one strand is shown. The complementary strand was also methylated at the CpG dinucleotide. Because of the symmetry of the sequence only the first half of the site needed to be analysed.

**Table 2:** A selection of p53 binding sites containing CpG dinucleotides from a data set of experimentally confirmed p53 binding sites as summarised^31^

Target gene and binding site	DNA sequence	Predicted log *K*_d_	Start position	Length	Positions of CpG	Δlog *K*_d_
Cell cycle						
Cyclin G	AGACCTGCCCGGGCAAGCCT	− 6.83	1	20	10	0
Cyclin G,C	AGGCTTGCCCGGGCAGGTCT	− 6.83	1	20	10	0
BTG2	AGTCCGGGCAGAGCCCGAGCA	− 4.94	1	20	5, 4	0^a^
GDF PTGF-b, SBS01 in Ref. 28	AGCCATGCCCGGGCAAGAAC	− 6.70	1	20	10	0
RB	GGGCGTGCCCCGCGTGCGCGCGC	− 6.40	1	23	4, 2, 4, 6, 10	− 0.42
Growth						
TGFA	AGCCAAGTCTTGGCAAGCGG	− 6.30	1	20	2	− 0.06
Death receptor						
TNFRSF Killer/DRS in Ref. 28	GGGCATGTCCGGGCAAGACG	− 7.09	1	20	1,10	0.04
PIDD PIDD in Ref. 28 LRDD in Ref. 29	AGGCCTGCCTGCGTGCTGGGACAAGTCT	− 6.75	1	28	Linker	0
DNA repair						
PCNA	ACATATGCCCGGACTTGTTC	− 6.83	1	20	10	0
Pcna	GAACAAGTCCGGGCATATGT	− 6.83	1	20	10	0
Apoptosis						
BAX-B,A	AGACAAGCCTGGGCGTGGGC	− 6.47	1	20	6	− 0.12
BAX-mouse	AGGCAAGCTTTGAACTTGCGG	− 6.67	1	21	2	− 0.06
BAX-human	GGGCAGGCCCGGGCTTGTCG	− 6.99	1	20	1, 10	0.04
IRDD cathepsin Dsite 1	AAGCTGGGCCGGGCTGACCC	− 5.56⁎	1	20	10	0
ei24/PIG8	TGGCAGGCCGGAGCTAGTTC	− 6.56	1	20	9	0
MCG10, RE-1 (PCBB4)	GAACTTAAGACCGAGGCTCTGGACAAGTTG	− 6.11	3	28	10	0
NOXA	AGGCTTGCCCCGGCAAGTTG	− 6.87	1	20	9	0
p53aip1	TCTCTTGCCCGGGCTTGTCG	− 6.73	1	20	1, 10	0.04
PERP, 2097	GCGCTAGTCCACACAGACTAGATT	− 6.33	1	24	2	− 0.06
cFOS,O	AGGCTTGCCCCGGCAAGTTG	− 6.87	1	20	9	0
Positive regulation						
mdm2 (13)	GGTCAAGTTGGGACACGTTC	− 6.66	1	20	4	− 0.12
S100A2	GGGCATGTGTGGGCACGTTC	− 6.93	1	20	4	− 0.12
Negative regulation						
cFOS,O	GGACTTGTCTGAGCGCGTGC	− 6.44	1	20	4, 6	− 0.24
Met	GGACGGACAGCACGCGAGGCAGACAGACACGTGC	− 6.20	8	27	4, 6, 8	− 0.34
Cytokeratin 8	CCGCCTGCCTCCACTCCTGCCT	− 5.85⁎	1	22	2	− 0.06
EEf1A1 EF-1 a E4 in Ref. 28	GGGCAGACCCGAGAGCATGCCC	− 6.61	1	22	10	0
EEf1A1 EF−1 a, E2 in Ref. 28	GGACACGTAGATTCGGGCAAGTCC	− 6.75	1	24	6, 10	− 0.12
EGFR	GAGCTAGACGTCCGGGCAGCCCC	− 5.81⁎	1	23	9, 10	0
Sgk	AACTCAGGCTGCCTCCTGCGACTTGCCT	− 6.26	2	27	9	0
TAP1	GGGCTTGGCCCTGCCGGGACTTGCCT	− 6.67	1	26	Linker	0
TIMP-3	GGGCTTGCTTGACGTCCAGAACAGGGTC	− 6.22	1	28	Linker	0
Human repressor						
ODC1	GGGCTCGCCTTGGTACAGACGAGCGGGCCC	− 6.16	1	30	6, 6, 10	− 0.24
ODC1	GGACCAGTTCCAGGCGGGCGAGACC	− 6.15	1	25	6, 10	− 0.12
slc38	AACCATGCTGTTACACGCACCAGCTTGTCC	− 6.58	11	20	6	− 0.12
p22/PRG1, IER3 in Ref. 29	CCACATGCCTCGACATGTGC	− 6.93	1	20	9	0
scd	GGGCCGGTCCTGGGCTAGGCT	− 5.92⁎	1	21	5	0.1
Hspa8	GCACTAGTTCTGGACCTCGCGCGTGCTT	− 6.22	1	28	6,8,10	− 0.22
NOS3	GAGCCTCCCAGCCGGGCTTGTTC	− 6.19	1	23	10	0

CpG dinucleotides are underlined. Sequences are presented in the same order. Please note that the exact arrangement of the binding site elements (i.e., location of the quarter-sites) is different from those presented for binding elements where the start position value is greater than one, as this arrangement was predicted to result in stronger binding. The position of CpG dinucleotide refers to the respective position in the half-site.

a Effects of individual CpG dinucleotides cancel each other.
